# 2240. Breaking Dogmas: A Single Center Evaluation of Empiric Vancomycin Use Among Intensive Care Unit Patients with Suspected Pneumonia, Bacteremia or Sepsis

**DOI:** 10.1093/ofid/ofad500.1862

**Published:** 2023-11-27

**Authors:** Christina Y Xie, Ashton Walsh, Maureen Campion, Kap Sum Foong, Shira Doron, Gabriela Andujar Vazquez

**Affiliations:** Tufts University School of Medicine, Diamond Bar, California; Tufts University, Seattle, Washington; Tufts Medical Center, Boston, Massachusetts; Tuft Medical Center, Boston, Massachusetts; Tufts Medical Center, Boston, Massachusetts; Tufts Medical Center, Boston, Massachusetts

## Abstract

**Background:**

Augmented concern for methicillin-resistant *Staphylococcus aureus* (*SA*) infections in intensive care units (ICU) has led to increased empiric vancomycin use (EVU) in patients with suspected pneumonia, bacteremia, and/or sepsis. At Tufts Medical Center, vancomycin is prescribed at clinician discretion without restrictions. We aimed to evaluate the impact of cultures on EVU and identify predictors associated with prolonged EVU among ICU patients with cultures negative (CN) for *SA.*

**Methods:**

A retrospective study from April 2022 to January 2023 of ICU adult patients with EVU for suspected pneumonia, bacteremia, or sepsis was conducted. ICU service, length of stay (LOS), duration of EVU, blood & respiratory cultures, mechanical ventilation (MV), central line presence, and vasopressor use were collected. Prolonged EVU was defined as vancomycin use for more than 2 days despite CN for *SA*. The primary outcome was the rate of prolonged EVU discontinuation. Linear regression included demographics, ICU service, LOS, indication, Charlson Co-morbidity Index, lactate, MV, and 0.5 mg/dL serum creatinine change. Logistic regression included the same variables plus age, positive respiratory cultures and MV interaction.

**Results:**

A total of 165 ICU patients had EVU. Of these, 132 (80%) had cultures collected before EVU vs. 33 (20%) that did not. Blood cultures were collected in 117 (88.6%); 66 (49.6%) had respiratory cultures; 51 (38.6%) had both collected. Median vancomycin duration was 2 days (1-3). Prolonged EVU was observed in 47 (46.1%) patients, while 55 (53.9%) had EVU discontinued within 2 days with CN for SA. On average Cardiac Surgery (CS) spent 2 days longer on vancomycin compared to Surgical (p=0.037) and Pulmonary (p=0.01) patients. MV among ICU patients was less likely to be associated with vancomycin discontinuation within 2 days with CN for SA (aOR [adjusted odd ratio] 0.12, 95% CI 0.03, 0.50; *p*=0.003).

**Conclusion:**

EVU discontinuation was more likely within 2 days in ICU patients with CN for SA, but less likely for MV or CS patients. In a substantial proportion of patients, cultures were not collected. Our findings highlight the importance of diagnostic testing in ICU settings to help reduce EVU.

**Disclosures:**

**Maureen Campion, PharmD**, Shinoigi: Speaker

